# Supplementary material for: Complexome profiling of the Chlamydomonas psb28 mutant reveals TEF5 as an early PSII assembly factor
Source: Plant Cell. 2025 Mar 17;37(6):koaf055. doi: 10.1093/plcell/koaf055 (PMC12164590; doi:10.1093/plcell/koaf055)
Supplement: koaf055_Supplementary_Data [file koaf055_supplementary_data.zip › psb28_interactive.zip!/plots/intensity/qualityPlots/peptides/CCB2_Cre12.g537850.t1.2.html]

### Cre12.g537850.t1.2

Trivial name: CCB2  
SeqCoverage [median]: 15.20  
Mass: 31.98 kDa  
  
 

| Sequence | MissedCleavage | Mass [Da] | wtIntensity | muIntensity | Score |
| --- | --- | --- | --- | --- | --- |
| DLSAVSGSSK | 0 | 949.47 | 2389.49 | 2853.93 | 101.64 |
| GALGSGVVAPGNAAASLAAMSK | 0 | 1898.99 | 633.01 | 3357.30 | 70.17 |
| GGLGDDDIDVAVFR | 0 | 1447.69 | 518.59 | 0.00 | 64.44 |
| RGGLGDDDIDVAVFR | 1 | 1603.80 | 0.00 | 2450.98 | 41.38 |
|  |  |  | 3541.1 | 8662.2 |  |
